# Single cell transcriptomic profiling of large intestinal enteroendocrine cells in mice – Identification of selective stimuli for insulin-like peptide-5 and glucagon-like peptide-1 co-expressing cells

**DOI:** 10.1016/j.molmet.2019.09.001

**Published:** 2019-09-07

**Authors:** Lawrence J. Billing, Pierre Larraufie, Jo Lewis, Andrew Leiter, Joyce Li, Brian Lam, Giles SH. Yeo, Deborah A. Goldspink, Richard G. Kay, Fiona M. Gribble, Frank Reimann

**Affiliations:** 1 University of Cambridge, Wellcome Trust/MRC Institute of Metabolic Science (IMS) & MRC Metabolic Diseases Unit, Addenbrooke's Hospital, Hills Road, Cambridge, CB2 0QQ, United Kingdom; 2 Division of Gastroenterology, Department of Medicine, University of Massachusetts Medical School, Worcester, MA, United States

**Keywords:** Single cell RNA-sequencing, Enteroendocrine cells, Glucagon-like peptide-1 (GLP-1), Insulin-like peptide-5 (Insl5), Serotonin (5-HT)

## Abstract

**Objective:**

Enteroendocrine cells (EECs) of the large intestine, found scattered in the epithelial layer, are known to express different hormones, with at least partial co-expression of different hormones in the same cell. Here we aimed to categorize colonic EECs and to identify possible targets for selective recruitment of hormones.

**Methods:**

Single cell RNA-sequencing of sorted enteroendocrine cells, using NeuroD1-Cre x Rosa26-EYFP mice, was used to cluster EECs from the colon and rectum according to their transcriptome. G-protein coupled receptors differentially expressed across clusters were identified, and, as a proof of principle, agonists of Agtr1a and Avpr1b were tested as candidate EEC secretagogues *in vitro* and *in vivo.*

**Results:**

EECs from the large intestine separated into 7 clear clusters, 4 expressing higher levels of *Tph1* (enzyme required for serotonin (5-HT) synthesis; enterochromaffin cells), 2 enriched for *Gcg* (encoding glucagon-like peptide-1, GLP-1, L-cells), and the 7th expressing somatostatin (D-cells). Restricted analysis of L-cells identified 4 L-cell sub-clusters, exhibiting differential expression of *Gcg*, *Pyy* (Peptide YY), *Nts* (neurotensin), *Insl5* (insulin-like peptide 5), *Cck* (cholecystokinin), and *Sct* (secretin). Expression profiles of L- and enterochromaffin cells revealed the clustering to represent gradients along the crypt-surface (cell maturation) and proximal-distal gut axes. Distal colonic/rectal L-cells differentially expressed *Agtr1a* and the ligand angiotensin II was shown to selectively increase GLP-1 and PYY release *in vitro* and GLP-1 *in vivo*.

**Conclusion:**

EECs in the large intestine exhibit differential expression gradients along the crypt-surface and proximal-distal axes. Distal L-cells can be differentially stimulated by targeting receptors such as Agtr1a.

## Highlights

• Large intestinal enteroendocrine cells group into subclusters by single cell RNAseq.
• Enteroendocrine-cell subclusters differ along crypt-surface and longitudinal axes.
• L-cells differ longitudinally by production of NTS (proximal colon) or INSL5 (rectum).
• INSL5-positive cells express distinct GPCRs enabling cluster-specific stimulation.
• Targeted stimulation of INSL5-producing L-cells elevates plasma GLP-1 and PYY *in vivo*.

## Graphical abstract

**Figure:**
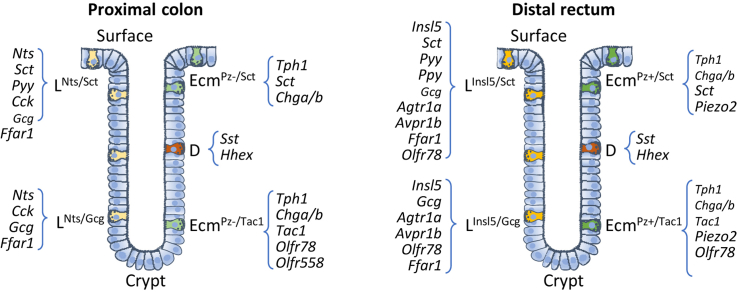

## 1 Introduction

Enteroendocrine cells (EECs) are a rare subset of gastrointestinal epithelial cells that regulate physiological processes including intestinal motility and secretion, glycemia, and appetite. They represent a diverse cellular population, collectively producing more than twenty different hormones [1], [2]. Gut hormone secretion after a meal is stimulated by nutrient absorption and is dominated by EECs from the small intestine, whereas the physiological role of the large number of EECs in the large intestine is less clear. However, EECs and the hormones they produce are candidate targets for drug development, as highlighted by the success of therapies based on Glucagon-like peptide-1 (GLP-1) for the treatment of type 2 diabetes and obesity. The aim of this project was to improve our understanding of the physiology of EECs in the large intestine (colon and rectum) and whether they could usefully be targeted therapeutically.

EEC populations vary along the length of the gastrointestinal (GI) tract, with some hormones produced predominantly in the proximal gut (e.g. Glucose-dependent insulinotropic polypeptide, GIP) and others predominating more distally (e.g. Peptide YY, PYY; GLP-1; Insulin-like peptide-5, INSL5) [3]. Recent transcriptomic analyses have challenged the traditional notion that distinct EEC subtypes exist, which produce separate and non-overlapping sets of gut hormones [4]. Characterization of individual EECs in the small intestine by single cell RNA-sequencing (scRNA-seq), led to the identification of distinct EEC subgroups by cluster analysis, exhibiting overlapping expression profiles for known gut hormones [5], [6], [7]. For example, Glass et al. found that subgroups of cells expressing *Gcg* (encoding GLP-1), classically known as L-cells, also expressed *Gip* (considered a product of K-cells) as well as *Tph1* (tryptophan hydroxylase-1), the enzyme required for serotonin (5-HT) production, implying overlap between L, K, and enterochromaffin (Ecm) cells [5]. Immunohistological and flow cytometric studies confirmed that these overlaps identified by transcriptomics were also reflected at the level of protein synthesis [8], [9], [10]. Most previous investigations, however, have focused on the small intestine rather than the colon.

In the large intestine, enterochromaffin cells have been reported as the most prevalent subtype of EEC [11]. These cells are defined by production of 5-HT, which exerts a critical role in regulating GI motility and peristalsis and has been associated both with irritable bowel syndrome (IBS) and inflammatory bowel disease (IBD) [12], [13]. L-cells are also highly abundant, and distinguishable by their production of GLP-1 and PYY, peptides known to suppress appetite and stimulate insulin secretion [11], [14], [15], [16], [17], [18], [19]. A third and rarer population known as D-cells produces somatostatin (SST) [11], which acts as a paracrine inhibitor of other EECs and excitatory cells and influences colonic motility [20], [21], [22], [23]. Recently, we showed that approximately half of all large intestinal L-cells produce INSL5, suggesting the existence of at least two subgroups of L-cells in this region [24], [25]. Expression of *Insl5* was restricted to the large intestine and absent in other regions of the GI tract. Large intestinal EECs are likely to sense different physiological stimuli compared with those located more proximally, as ingested nutrients do not normally reach the distal gut in high quantities, and resident microbiota produce a variety of alternative candidate signaling molecules.

EECs are generated alongside other intestinal epithelial cells by the continuous division of crypt stem cells, and in the duodenum and jejunum have been reported to have a life span of 3–10 days before they are shed into the lumen from the villus tips [26], [27], although a recent paper has shown longer life spans of EECs compared to surrounding enterocytes in the small intestine [28]. Small intestinal EEC development and maturation has been modeled using 3-dimensional intestinal organoid cultures, revealing that L-cells and Ecm cells mature as they migrate from crypts into villi, developing increased expression of *Sct* (secretin), accompanied by reductions of *Gcg* expression in L-cells and of *Tac1* (tachykinin) in Ecm cells [7], [28]. Large intestinal epithelium, by contrast, is characterized by deep crypts and no villi, and reports that EECs in this region have longer life spans of about three weeks [29] suggest some differences in EEC maturation compared with the small intestine.

In this study, we mapped large intestinal EECs cells using single cell RNA-sequencing. We identified different subpopulations of L-cells and Ecm-cells, and showed that these likely represent cellular gradients mapping along the proximal-distal and crypt-surface gut axes. Selective stimulation of distal L-cells using Angiotensin-II resulted in significant elevation of plasma GLP-1 levels, suggesting that these cells can contribute to circulating gut hormone concentrations despite their distal location.

## 2 Methods

### 2.1 Animal work and ethics

All animal procedures were approved by the University of Cambridge Animal Welfare and Ethical Review Body and carried out in accordance with the Animals (Scientific Procedures) Act 1986 Amendment Regulations (SI 2012/3039). The animal work was performed under the UK Home Office project licences 70/7824 and PE5OF6065 [30], [31]. Mice were housed in ventilated cages on a 12hr light/dark cycle (lights out at 07:00 GMT) with ad libitum access to water and regular chow (unless otherwise stated) and were culled by an approved Schedule 1 method.

### 2.2 Flow cytometry

Single cell digests of mouse colon were prepared for FACS purification by two incubations of 30 min with 1 mg/ml collagenase (dissolved in calcium-free HBSS) at 37 °C. After each incubation, single cells were harvested in the media, washed in calcium-free HBSS with 10% FBS and filtrated through 50 μm filters. The two digests were spun at 300 g for 10 min at 4 °C and resuspended together in HBSS (calcium-free) with 10% FBS and stained 5 min on ice with DAPI (1 μg/mL) and washed once before being resuspended in HBSS with 10% FBS, 10 μM Y-27632 and 5 μM Draq5 (Biolegend)

The single cell suspension was sorted using an Influx Cell Sorter (BD Biosciences) at the Cambridge Institute of Medical Research (CIMR) Flow Cytometry Core Facility. DAPI-staining, DRAQ5-staining, side scatter, forward scatter and pulse width gates were applied to remove clustered cells, dead cells and cellular debris present. *Neurod1*-expressing cells (from a NeuroD1-Cre x Rosa26-EYFP mouse, henceforth called NeuroD1^EYFP^) were purified by EYFP fluorescence into LoBind tubes (Eppendorf) with 40 μl HBSS (calcium-free), 10% FBS and 10 μM Y-27632 [30], [31].

### 2.3 Single-cell RNA-sequencing

#### 2.3.1 Library preparation and sequencing

3500 FACS-purified NeuroD1^EYFP^ cells (from a single mouse colon) were loaded onto the Chromium system (10x Genomics 3′ GEX V2) to produce cDNA libraries, which were paired-end sequenced (26:8:98) by a HiSeq 4000 (Illumina) at the Cancer Research UK Cambridge Institute (CRUK CI). Quality control, read alignment (with reference to the mm10 genome downloaded from the UCSC genome browser [32]) and raw count quantification for each cell was achieved using the CellRanger pipeline (10x Genomics).

#### 2.3.2 scRNA-seq analysis

Analyses from raw counts were performed using the Seurat package (v2.3.4, Butler et al., 2018 Nat biotechnologies) in R using default parameters except when indicated. Cells were first filtered based on their total number of expressed genes (min = 800), nUMI (unique molecular identifiers; min = 1250, max = 3rd quartile + interquartile) and the percentage of mitochondrial genes (between 1 and 7.5%). A first analysis was performed using standard normalization to retrieve the list of genes that are differently expressed (DE) in at least one population. As DE genes accounted for a majority proportion of UMIs, cells were further filtered on their number of UMI matching DE based on a first analysis with standard normalization (min 800). Moreover, UMI normalization was performed based on the number of UMI matching nonDE genes rather than the total number of UMIs per cell. The total number of cells of filtering was 1560.

Clusters were identified by shared nearest neighbor clustering optimization using the 7 most variable dimensions of a principal component analysis performed on the most variable genes. Populations were plotted using t-SNE dimension reduction or expression of specific genes plotted on distribution plots or heatmaps. Differentially expressed genes (FDR of 5%) were assessed using a Wilcoxon rank test between individual populations against the rest of the cells or between two specific populations or groups of populations with a log-two-fold difference between each group of at least 0.2.

Subpopulations were analyzed by subsetting the cells based on cluster annotation during first analysis and performed as the general one, using appropriate numbers of dimensions when identifying the clusters.

### 2.4 Real-time quantitative PCR (RT-qPCR)

Tissue samples were harvested from the colon divided into seven equal parts along the proximo-distal axis (P1–P7) and lysed using TRI-reagent. RNA was extracted by adding chloroform and collecting the aqueous phase while proteins and peptides were retained in the phenol phase. RNA was purified by isopropanol and ethanol precipitation and resuspended in nuclease free water and treated with DNA-free DNA removal kit (Invitrogen) to remove residual genomic DNA. RNA was reverse transcribed using SuperScript II using a Peltier Thermal Cycler-225 (MJ Research) according to standard protocols. The RT-qPCR reaction mix consisted of template cDNA, TaqMan Universal Master Mix (Applied Biosystems) and specific primers (Applied Biosystems) for β*-actin* (Mm02619580_g1), *Insl5* (Mm00442241_m1), *Gcg* (Mm01269055_m1), *Pyy* (Mm00520716_g1), *Nts* (Mm00481140_m1), *Cck* (Mm00446170_m1), *Tph1* (Mm01202614_m1), *Sct* (Mm00441235_g1), *Sst* (Mm00436671_m1), *Tac1* (Mm01166996_m1), Piezo2 (Mm01265858_m1), Ffar1 (Mm00809442_s1), *Agtr1a* (Mm01957722_s1) and *Avpr1b* (Mm01700416_m1) and qPCR were performed and analyzed using an ABI QuantStudio 7 (Applied Biosystems). Relative expression was evaluated by calculating the difference in cycle threshold (ΔCT) between the housekeeper gene β*-actin* and the gene of interest (CT_β-actin_-CT_Gene_).

### 2.5 Peptide extraction

Proteins were extracted from the phenol phase (after DNA precipitation by ethanol) by incubation with ice-cold acetone for 10 min at 4 °C followed by centrifugation for 10 min, 12000 g. Following a wash in 0.3 M guanidine HCl (dissolved in 95% ethanol), the resulting pellet was air dried and resuspended in 8 M urea using a syringe. Then 80% acetonitrile was then added to each sample to precipitate proteins and peptides in solution recovered and dried using a centrifugal concentrator [3]. Peptides were resuspended in 500 μL 0.1% formic acid and spiked with internal standards and purified by solid phase extraction using a prime HLB μelution plate (Oasis) and eluted in 60 μL 60% methanol, 30% H_2_O, and 10% acetic acid. Peptides were dried and reduced-alkylated by incubation 1 h with 10 mM DTT in 50 mM ammonium bicarbonate at 60 °C followed by 30 min incubation with 20 mM iodoacetamide. Samples were further diluted with 0.1% formic acid and 10 μL were analyzed using a nano-flow rate by a ThermoFisher Ultimate 3000 nano LC system coupled to a Q Exactive Plus Orbitrap mass spectrometer (Thermo Scientific) [3], [33]. Peptide quantification was achieved using XCalibur (ThermoFisher) to integrate the peak area for selected sets of *m*/*z* values at specific retention times for each individual peptide.

For the LC-MS/MS peptide quantification, treatment supernatants were first acidified with 50 μl 1% formic acid while homogenates were resuspended in 500 μl 0.1% formic acid. Both were subsequently spiked with internal standards. Subsequently peptides were extracted and analyzed following reduction/alkylation as described previously [3], [34]. Total protein content was calculated from lysate supernatants using a BCA protein assay (Thermo Fisher Scientific) which was used to normalize secretory responses from different wells. In several cases surrogate peptides, chosen on the basis of their robust observable signal, are reported and considered to be produced from the pro-peptide in a stable molar ratio to the established hormones. For Gcg, we chose oxyntomodulin (Figure 3) and GRPP (Figure 4) as the GLP-1 signal was low and split between amidated and non-amidated forms; for CCK we chose CCK21_44, as sulfated CCK8 was not detectable in the LC-MS/MS mode needed for the other peptides; for INSL5, we chose the C-chain.

### 2.6 Immunohistochemistry

Colonic wholemounts were processed using adapted methods detailed by Winton et al. (1990) [35]. Briefly, following isolation of the epithelial layer, the colons were fixed for 3 h at room temperature using 4% PFA (Alfa Aesar). Next, residual mucus was removed from the fixed tissue by incubation with 50 ml of demucifying solution (10% glycerol, 10% 0.1 M Tris titrated to pH 8.2, 20% ethanol, 92 mM NaCl, and 20 mM DTT) for 20 min at room temperature followed by PBS washes. Afterward, the fixed colons were placed in blocking solution (PBS with 0.1% Triton-X 100 and 10% goat serum) overnight at 4 °C. The next day, the colons were incubated for 4 h at room temperature with primary antibodies to PYY (guinea pig; Progen 16066; 1:500), INSL5 (rat; Takeda; 1:1000), and NTS (rabbit; Merck; AB4596; 1:100) diluted in wash solution containing 1% goat serum and 0.1% Triton-X 100 in PBS. Tissues were washed overnight and then incubated with 1:300 goat secondary antibodies (conjugated to AlexFluor 488, 555 and 633) for 3 h at room temperature. Following further washing overnight at 4 °C, the colons were incubated with 1:2000 Hoescht nuclear stain (in PBS) for 30 min at room temperature followed by PBS washes. Finally, the colons were divided in half and mounted onto microscope slides using Hydromount (National Diagnostics).

### 2.7 Imaging of colonic wholemounts

#### 2.7.1 Image acquisition

Wholemounts were imaged using the Axio Scan.Z1 system (Zeiss). Tiles of extended depth of focus (EDF) images were taken for each of the three labeled channels using a Plan-ApoChromat 20x/0.8 M27 objective, a Hamamatsu Orca Flash camera, and an inbuilt autofocus function. The depths used for the EDF images were customized for each wholemount and depended on tissue thickness. Following acquisition, the tiled images were stitched together with shading correction.

#### 2.7.2 Counting of immunofluorescently labeled cells

For analysis of cell populations from stained whole-mount tissue, 10 ROIs (0.5 mm × 0.5 mm picked based on in focus Hoescht staining) where selected from proximal, mid and distal areas of the large intestine from 3 mice (total ROIs = 90) and the number of cells for each population counted using HALO software (Indica Labs). For automated analysis, thresholding and size criteria were kept the same for all 3 channels and the number of cells per ROI for each of the channels was then calculated. Cell density per region was then analyzed using a combination of Excel (Microsoft Office) and GraphPad Prism 7.0 (GraphPad Software).

### 2.8 Primary cultures

Excised murine large intestines were collected in Leibovitz's L-15 media and divided into three equal segments. Segments from the same region from 2 different mice were pooled together to ensure enough tissue for each secretion plate. Colonic crypts were subsequently isolated from each region as described previously [36]. Briefly, isolated tissue was cleaned of contents in PBS containing CaCl_2_ and MgCl_2_ and the epithelium was separated from adipose, the outer muscle layers and vasculature by manual stripping. Following this, the tissue was cut into small chunks (∼2 mm^2^) and digested using collagenase XI (Sigma; 0.25 mg/ml). Isolated colonic crypts were resuspended in DMEM enriched with glucose (4500 mg/L), 10% FBS, 1% glutamine, and 1% penicillin/streptomycin and plated onto 2% matrigel (Corning) precoated 12 well plates. Plated crypt suspensions were placed into a 37 °C humidified incubator with 5% CO_2_ to settle overnight prior to experimentation.

### 2.9 Secretion assays

For the secretion assays, colonic crypt cultures were processed as described in Billing et al. [25]. Briefly, following washes with saline buffer containing 1 mM glucose and 0.001% fatty acid free BSA, each well of colonic crypts was incubated with 600 μl of each treatment (made up in saline buffer and 0.001% BSA) at 37 °C for 1 h. Supernatants were collected in protein LoBind 1.5 ml tubes (Eppendorf) and centrifuged at 2000 g for 5 min at 4 °C to remove cellular debris. 500 μl of each supernatant were collected into fresh 1.5 ml LoBind tubes. Meanwhile, 200 μl lysis buffer was added to each well. After 30 min on ice, the plates were snap frozen and defrosted to ensure complete cell lysis. Lysates were collected following cell scraping and spun at 2000 g for 5 min at 4 °C and the supernatants were retained. Both supernatants and lysates were stored at −80 °C prior to further processing. Protein content from lysates was analyzed by BCA assay (Pierce) and used to normalize for cell density variability between wells. 100 μL 1% formic acid and internal standards were added to supernatants and peptides were extracted by solid phase extraction and reduced alkylated as described previously. 40 μL of 120 μL were analyzed by nano-LC-MS/MS as described previously and peptide content quantified by measuring the peak area corresponding to characterized peptides. The experiment was performed five times with duplicates for each condition. Each peptide was analyzed separately and two-way ANOVA followed by a Tukey's test were performed to test significant differences between responses.

### 2.10 *In vivo* stimulation test

Adult male and female mice, obtained from a C57Bl6 colony maintained at the University of Cambridge, were fasted overnight (16 h) with free access to water. AVP (V9879, Sigma - 100 ng per mouse), angiotensin II (A9525, Sigma - 1 mg/kg BW) or vehicle (saline) were administered via intraperitoneal injection. For measurement of GLP-1 (cross-over design) and PYY levels (independent experiment, due to the greater plasma volume required for the assay), 50 and 80 μl of blood were collected from the tail vein into heparin-coated tubes 15 min post administration. Plasma was separated by centrifugation at 4 °C and snap frozen on dry ice, before storage at −80 °C. Plasma GLP-1 and PYY were measured in 20 and 40 μl respectively by non-competitive two-site immunoassay (MesoScale Discovery/CBAL UK). Data were analyzed by Student's paired and unpaired t-test for GLP-1 and PYY, respectively.

## 3 Results

### 3.1 EEC heterogeneity in the colon

**Figure 1:**
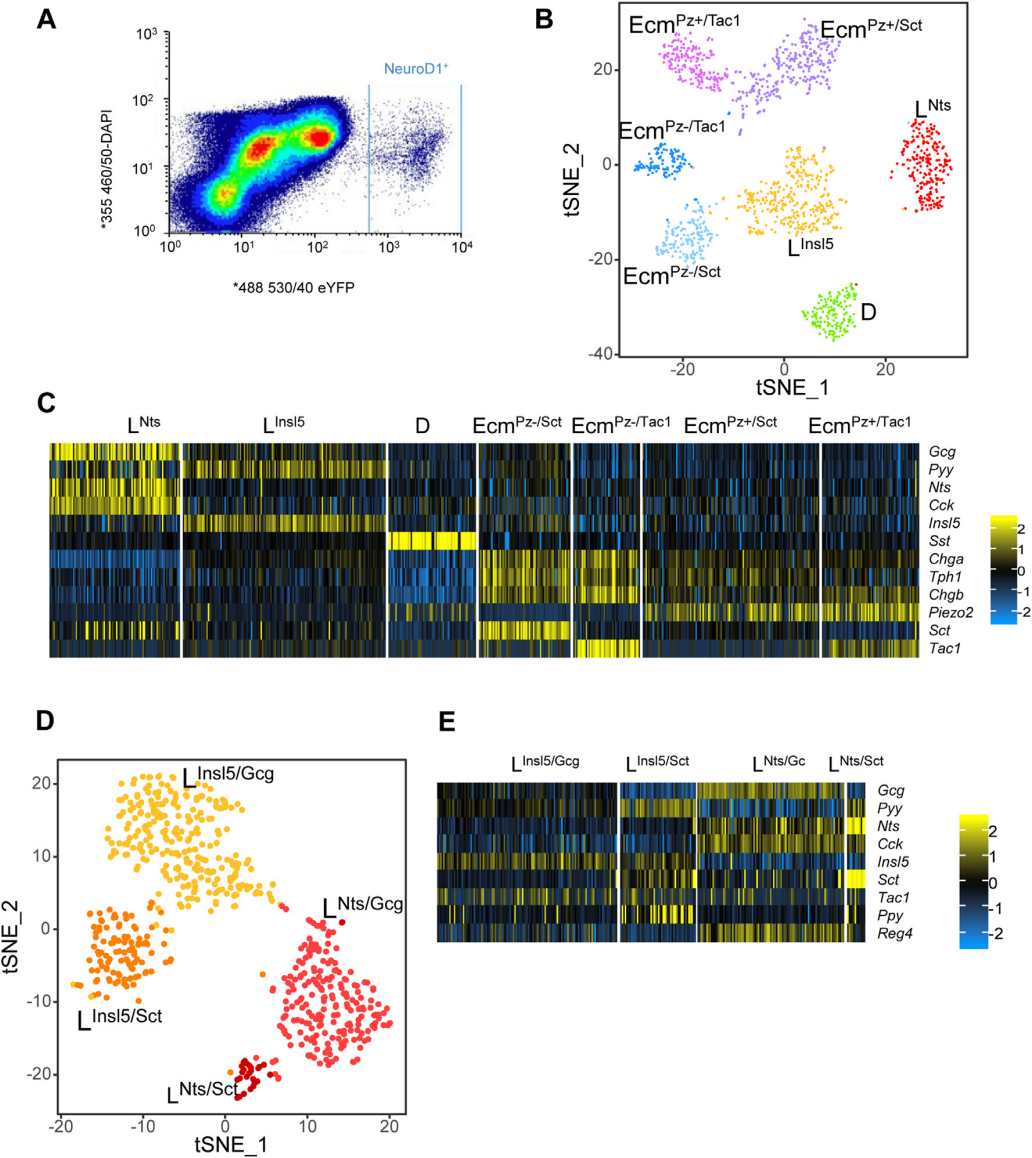
**Identification of colonic enteroendocrine cell clusters.** (A) FACS output from the NeuroD1 sort. Sorted fluorescent EYFP cells ∼0.58% of parent population following forward/side scatter, DRAQ5 and DAPI gating. 7000 EYFP^+ve^ cells were isolated in one FACS run from one NeuroD1-EYFP mouse. 3500 of these cells were put into the Chromium 10x system. (B) t-SNE plot of identified cell clusters from scRNA-seq analysis of FACS-isolated NeuroD1 expressing colonic cells (n = 1560 cells). Clusters were identified from the first 7 dimensions of a PCA analysis of the most variable genes using a shared nearest neighbour algorithm. (C) Heat-map of relative expression to average for the main hormone encoding genes and markers of the Nts and Insl5 L-cell sub-clusters, D-cells, Secretin, Tac1 and Piezo2 Ecm cell sub-clusters. (D) t-SNE plot of all cells identified as L-cells from the first clustering, with the clusters identified using an SNN algorithm on the 7 first dimensions. (E) Heat-map of relative expression to average for the main hormone encoding genes of the L-cell sub-clusters.

EECs from a Neurod1-cre mouse crossed with a Rosa26-EYFP reporter mouse colon were flow-sorted and single-cell RNA-seq was performed using the 10xGenomics 3′ GEX V2 platform (see methods) (Figure 1A). Data were analyzed using the Seurat R package with modifications as described in material and methods. Cluster analysis identified 7 EEC subgroups (Figure 1B). Gut hormones were amongst the top differentially expressed genes distinguishing clusters, including *Gcg*, *Nts* (neurotensin), *Insl5*, *Pyy*, *Cck* (cholecystokinin), *Sst*, *Sct, Tac1*, and *Tph1* (Suppl. Figure S1A–C and Figure 1C). Four clusters expressed high levels of *Tph1*, identifying them as Ecm cells (790/1560 cells, ∼50%), two clusters were enriched for *Gcg* and *Pyy* characteristic of L-cells (609/1560 cells, ∼40%), and the remaining cluster expressed high levels of *Sst* (D-cells, 161/1560 cells, ∼10%) (Figure 1C). Each of the clusters expressed specific set of genes with the top5 being presented in Suppl. Figure S1A.

Two of the four Ecm-cell clusters showed enrichment for the expression of the mechanosensitive ion channel *Piezo2* and have been denoted as Ecm^Pz+^, with the two Piezo2-negative clusters denoted Ecm^Pz−^. Both *Piezo*-positive and *Piezo*-negative cells sub-clustered into groups that were enriched for either *Sct* or *Tac1*, resulting in clusters labeled Ecm^Pz+/Sct^, Ecm^Pz+/Tac^, Ecm^Pz−/Sct^, and Ecm^Pz−/Tac^ (Figure 1C).

Comparing the two L-cell clusters, one expressed high levels of *Nts* and *Cck* (denoted L^Nts^-cells) whereas the other had high levels of *Insl5* (L^Insl5^-cells) (Figure 1C). When the L-cell clusters were further analyzed after exclusion of all other EECs, additional sub-clustering was observed (Figure 1D and Suppl. Figure S1D). L^Nts^-cells separated into two groups, one with higher *Nts*, *Pyy*, and *Sct* (L^Nts/Sct^) and the other with higher *Gcg* (L^Nts/Gcg^). L^Insl5^ cells similarly separated into a group with higher *Pyy* and *Sct* (L^Insl5/Sct^) and one with higher *Gcg* (L^Insl5/Gcg^) (Figure 1E).

Analysis of the D-cell cluster without other EEC sub-types did not reveal further D-cell sub-clusters. Differential expression heatmaps of G-protein coupled receptors (GPCRs) and transcription factors across all 7 EEC clusters, as well as across the four L-cell sub-clusters analyzed separately, are shown in Suppl. Figure S1.

### 3.2 Common transcriptomic patterns defining different EEC subgroups

**Figure 2:**
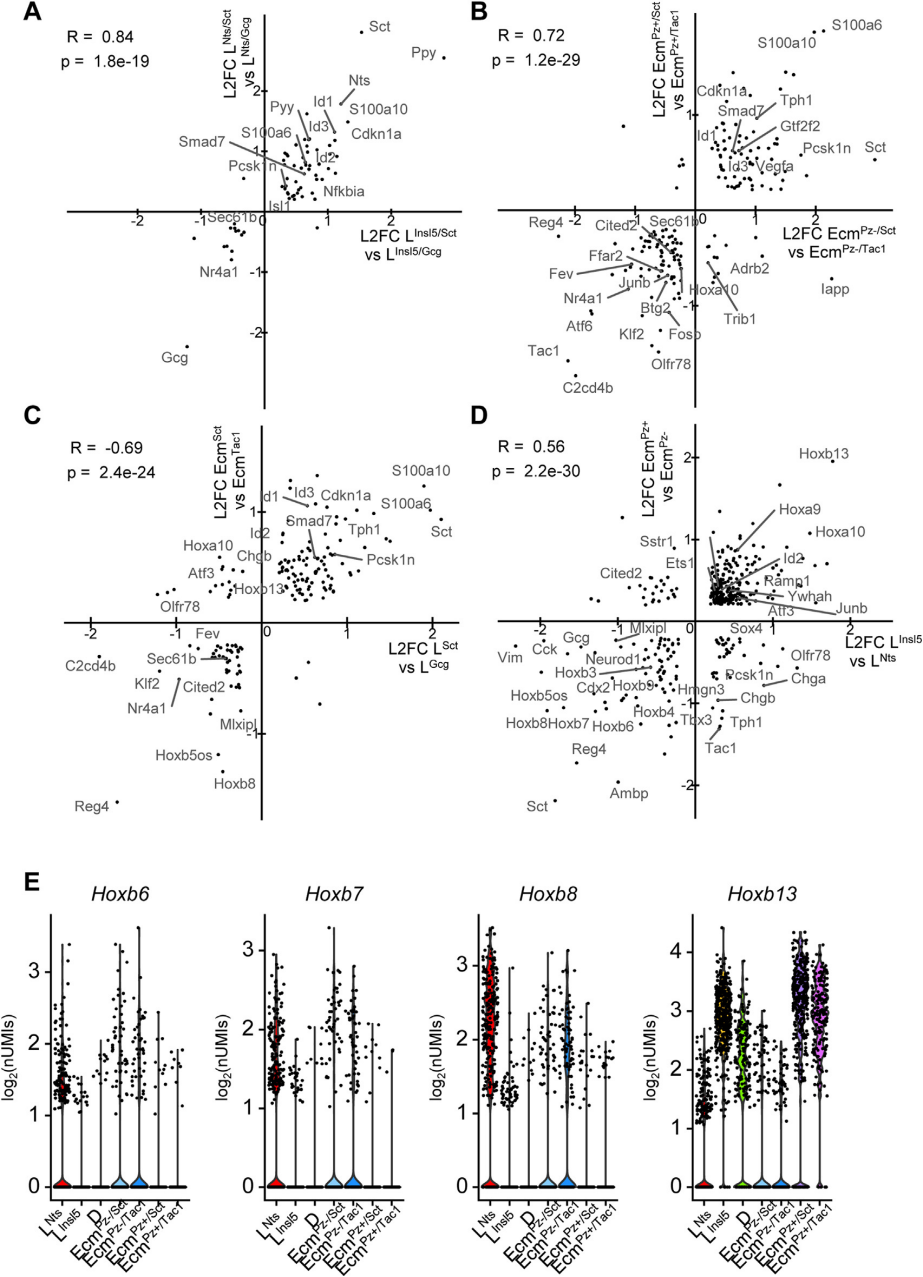
**Common determinants of Ecm- and L-cell sub-clustering**. (A–D) Correlation plots of all genes that are differently expressed in at least one comparison and for which the L2FC is higher than 0.2 in both comparisons, plotting the log_2_ fold change difference between two populations plotted against the log_2_ fold change between two other populations. Pearson correlation coefficient is indicated. (A) compares the difference between the L^Insl5/Gcg^ and the L^Insl5/Sct^ sub-clusters with the difference between the L^Nts/Gcg^ and L^Nts/Sct^ sub-clusters, as defined during the L-cells sub-clustering. (B) compares the difference between the Ecm^Pz−/Tac^ cluster and the Ecm^Pz−/Sct^ cluster with the difference between the Ecm^Pz+/Tac^ and the Ecm^Pz+/Sct^ clusters. (C) compares the difference between Sct-positive (L^Nts/Sct^ + L^Insl5/Sct^) and Sct-negative (L^Nts/Gcg^ + L^Insl5/Gcg^) L-cells with the difference between similar Ecm sub-clusters ((EC^Pz−/Sct^ + EC^Pz+/Sct^) and (EC^Pz−/Tac^ + EC^Pz+/Tac^), respectively). (D) compares the difference between Nts-positive (L^Nts^) and Insl5-positive (L^Insl5^) L-cells with the differences between Piezo-negative (EC^Pz−/Tac^ + EC^Pz−/Sct^) and -positive (EC^Pz+/Tac^ + EC^Pz+/Sct^) Ecm-cells. (E) Violin plots of log_2_ normalized unique molecular identifiers (nUMIs) counts for the original identified seven clusters for different Hoxb genes.

We hypothesized that our observation of Ecm and L-cell clusters with differential expression of *Sct*, *Tac1* (in Ecm cells), and *Gcg* (in L-cells) reflects cellular maturation along the crypt-surface axis in the colon and rectum, mirroring the recently described maturation of small intestinal EECs [7], [28]. We examined whether the acquisition of *Sct* expression in L-cells is accompanied by other transcriptional changes that are common between L^Nts^ and L^Insl5^ cells (Figure 2A), restricting our analysis to genes showing an absolute log_2_ fold-change (L2FC) of >0.2 in both *Sct*/*Gcg* pairs and reaching a significant difference in at least one of the *Sct*/*Gcg* pairs. Genes enriched in L^Nts/Sct^ cells (vs L^Nts/Gcg^) correlated positively with those enriched in L^Insl5/Sct^ cells (vs L^Insl5/Gcg^), with *Sct* and *Gcg* exhibiting the most extreme regulation between these clusters. Other genes highlighted by the analysis included the transcription factors *Id* (inhibitor of differentiation) *-1,2,3*, which were higher in *Sct*-enriched clusters, and *Nr4a1*, which was higher in *Gcg*-enriched clusters.

We performed a similar analysis of Ecm cell clusters to examine whether the acquisition of *Sct* and loss of *Tac1* expression is accompanied by other common transcriptional changes between *Piezo2*-positive and negative Ecm clusters (Figure 2B). This comparison showed reasonable correlation, with several genes being up- and down-regulated in parallel with *Sct* and *Tac1*. Genes enriched in the *Sct*-groups (*Cdkn1a*, *Smad7*) have been described as markers of colonic surface epithelium, whereas genes in the *Tac1*-cluster (*Sec61b, Atf6*) have been located towards the bottom of colonic crypts [37], consistent with the idea that downregulation of *Tac1* expression and upregulation of *Sct* occurs during Ecm cell maturation in the colon and rectum. A similar analysis comparing all L- and Ecm-cells, each grouped by their Sct-expression status, also showed a reasonable correlation, revealing an overlapping set of genes (Figure 2C) suggesting that these genes are commonly regulated along the crypt-surface epithelial axis in colonic EECs.

We next examined, independent of these maturity markers, what separated L-cells into high *Insl5* vs high *Nts* groups and Ecm cells into *Piezo2*-positive vs *Piezo2*-negative groups (Figure 2D). A differential expression analysis, performed as above but comparing genes enriched in *Piezo2* positive (vs negative) Ecm cells with those enriched in *Insl5*-positive L-cells (vs L^Nts^ cells), revealed parallel transcriptional changes in Ecm^Pz+^ cells and L^Insl5^ cells. This comparison identified a number of Homeobox B (*Hoxb*) genes which are known to be involved in rostro-caudal differentiation thereby defining the proximal to distal axis in the large intestine, with higher *Hoxb* numbers assigned to more distal locations [38], [39]. *Hoxb6*, *Hoxb7*, and *Hoxb8* were higher in Ecm^Pz−^ and L^Nts^ cells, suggesting they arise more proximally in the large intestine, whereas *Hoxb13* was higher in Ecm^Pz+^ and L^Insl5^ cells, suggesting a more distal origin (Figure 2E).

### 3.3 EEC variability along the proximo-distal axis

**Figure 3:**
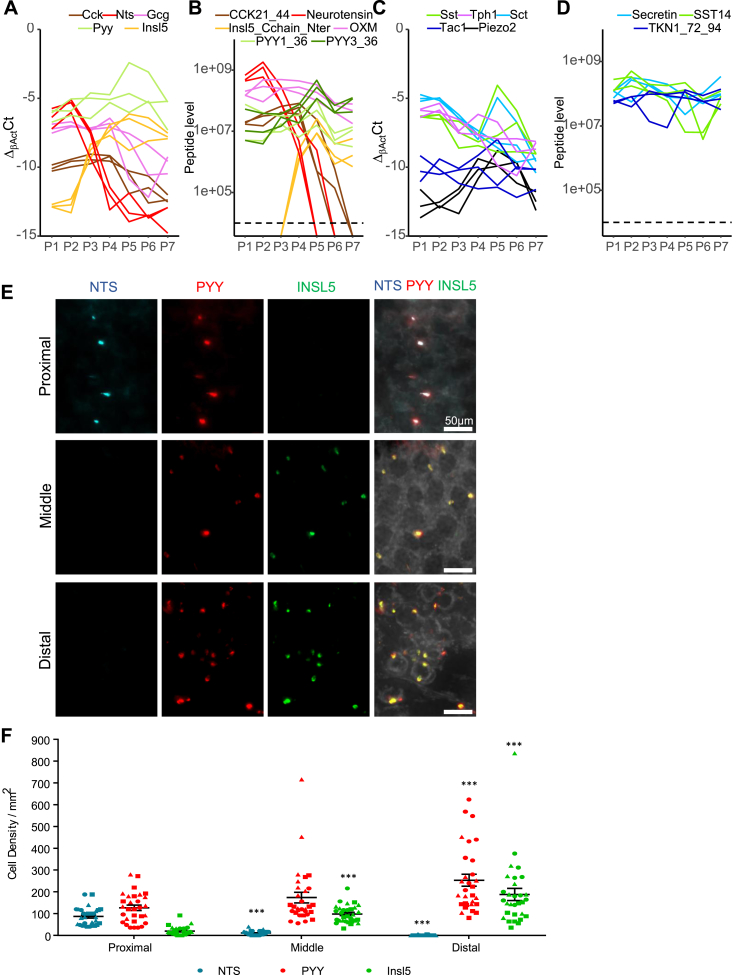
**Localization of INSL5, PYY, and NTS-producing cells within the colon**. (A, C) Relative expression of Lcell (A) and D or Ecm-cell (C) enriched genes along the proximal-distal axis of the murine colon divided into 7 equal segments (P1–P7) by RT-qPCR. Cycle threshold difference (ΔCT) was calculated between the gene of interest and the housekeeper β-actin (CT_β-actin_-CT_Gene_). (B,D) Peptide quantification by LC-MS/MS of proCCK (CCK21-44), Neurotensin, INSL5 (C-chain), Oxyntomodulin (OXM), PYY (1-36 and 3-36) (B) and pro-SST, pro-tachykinin (TKN)1 and SCT (D) in P1–P7. (E) Representative images of proximal, middle and distal large intestine immunofluorescently labeled for NTS (blue, left column), PYY (red, middle center column) and INSL5 (green, center right column). Merged pictures for all three regions are represented in most right column. Scale bar = 50 μm. (F) Plot showing the density (per mm^2^) of labeled NTS (blue), PYY (red) and INSL5 (green) cells in proximal, middle, and distal large intestine as extracted from images as shown in (D). Data from 3 mice (indicated by different symbols), with 10 ROIs per region per mouse. Analysis by non-parametric one-way ANOVA with post hoc Dunns multiple comparison (compared to proximal density) for each of the hormones. ***p < 0.001.

To confirm the proximo-distal distribution of the different clusters, gene expression and peptide levels were measured in tissue homogenates from seven regions equally distributed along the colon/rectum, from proximal (P1) to distal (P7) in three mice. *Insl5* gene expression was very low and the peptide undetectable in proximal regions whereas expression and INSL5 peptide levels were significantly increased in the distal gut (Figure 3A and B). *Nts* and *Cck* showed the opposite pattern, with high levels in proximal regions that dropped more distally. *Gcg* and *Pyy* (and derived peptides) only exhibited weak proximal-distal gradients along the large intestine, but, interestingly, we detected PYY3-36 as well as PYY1-36 by our LC-MS/MS analysis, and found that PYY3-36 predominated in the distal rectum, indicating region-dependent processing. We further validated the regional distribution of L-cells by co-staining for INSL5, NTS, and PYY in whole mounted colons (Figure 3E). NTS positive cells were mainly localized in the proximal regions whereas INSL5 positive cells were found in higher numbers in the distal colon (Figure 3F). PYY positive cells were present along the large intestine, with no evident gradient.

Consistent with the proposed distal location of Ecm^Pz+^ cells determined from the cluster analysis, *Piezo2* expression was 5 times higher in the distal tissue samples, with the exception of the most distal rectum. Gradients for other examined Ecm cell markers and *Sst* were unremarkable (Figure 3C and D).

### 3.4 Selective stimulation of L-cell populations by GPCR ligands

**Figure 4:**
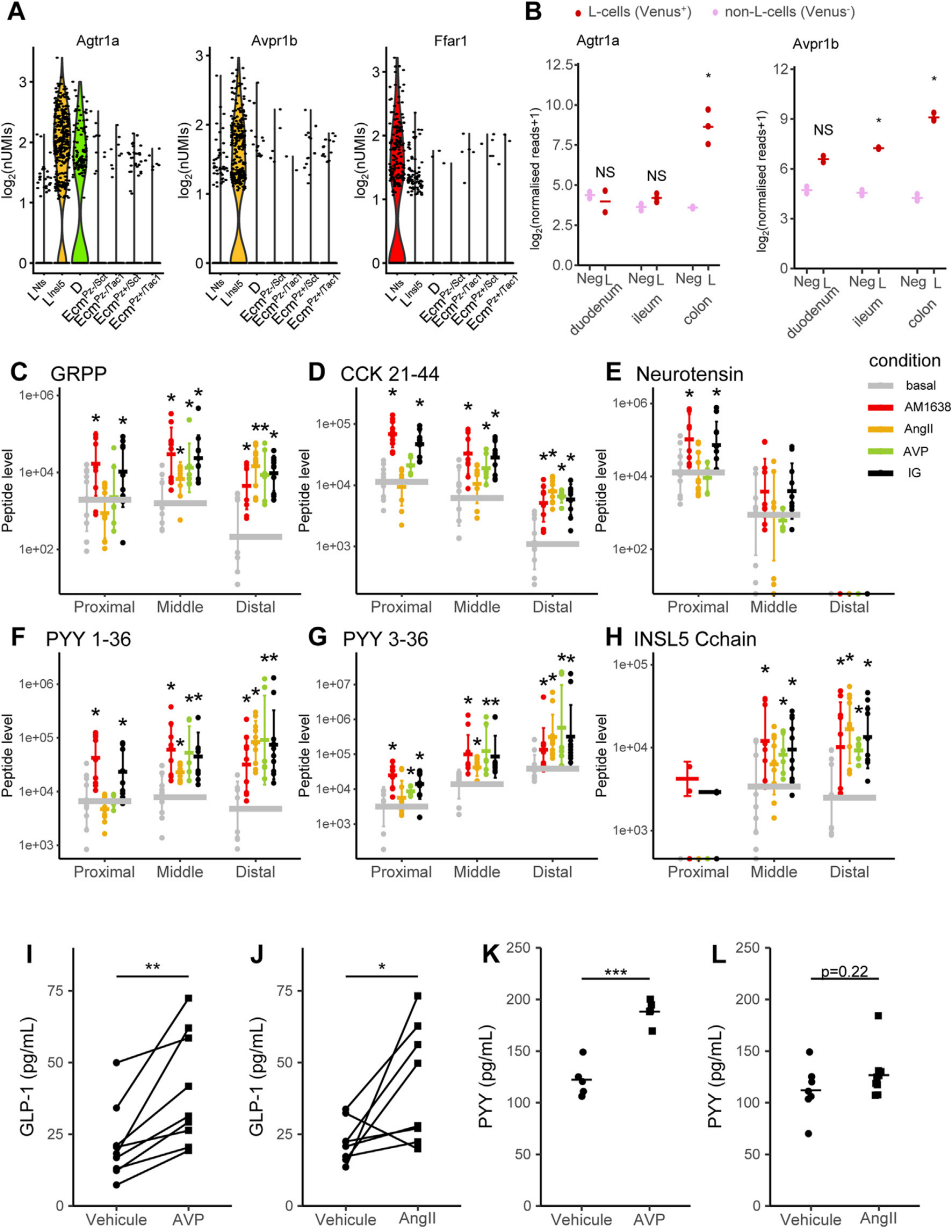
**Selective stimulation of distal colonic/rectal L-cells**. (A) Violin plots of log_2_ normalized unique molecular identifiers (nUMIs) counts in the seven colonic EEC clusters for Angiotensin-II receptor 1a (Agtr1a), Arginine-vasopressin receptor 1b (Avpr1b) and Free-fatty-acid receptor 1 (Ffar1). (B) Expression of Agtr1a and Avpr1b in Venus-labeled L-cells and non-fluorescent cells from the same sorts isolated from the duodenum, ileum, and colon from GLU-Venus mice; data from bulk RNAseq by Roberts and collaborators [3], shown as Log_2_ (normalized reads+1) using DESeq2 normalization, data for L-cells are shown in dark red and for negative cells in light red. (C–H) Secretion of different gut peptides as indicated in response to a FFAR1- (AM1638, 1 μM), Agtr1a- (AngII, 10 nM), and Avpr1b- (AVP, 10 nM) agonists or no stimuli or IBMX 100 μM with 10 mM glucose (IG). Colonic primary cultures from the first, middle and final third of mouse large intestine were processed separately and stimulated in parallel. Peptides were quantified by LC-MS/MS and normalized to the protein content of the crypt culture. * indicates a statistical difference between the condition and the basal (control condition) tested by a Tukey's test if a two-way ANOVA showed different populations. (I–L) Plasma GLP-1 (I, J) and PYY (K, L) levels 15 min after i.p. vehicle, AngII (1 mg/kg) (I, K) or AVP (100 ng/mouse) (J, L) application. Data were analyzed by Student's paired t-test for GLP-1 as mice were their own controls (cross over design) and by Student's unpaired t-test for PYY as measurements were performed in independent experiments. *p < 0.05, **p < 0.01 and ***p < 0.001.

As expression of some GPCRs was found to differ between the L-cell clusters (Supp. Figure S1E), we examined whether selective GPCR agonists could be used to trigger region-specific hormone secretion from the colon or rectum. We selected the arginine-vasopressin (AVP) receptor *Avpr1b* and the angiotensin2 (AngII) receptor *Agtr1a*, which were enriched in L^Insl5^ compared with L^Nts^ cells, and the free fatty acid receptor *Ffar1*, which was expressed in both L-cell clusters (Figure 4A) but at lower level. Primary crypt cultures from the proximal third, the middle third or the distal third (Figure 4C–H) of the large intestine were stimulated with AVP (10 nM), AngII (10 nM) or AM1638 (1 μM; a strong agonist for FFA1). Hormone secretion was quantified by a multiplex LC-MS/MS method [25], [34], and a combination of glucose (10 mM) and IBMX (100 μM) was used as a positive control. We were unable to detect secretin reliably with this method, perhaps reflecting the enrichment of crypt over surface epithelial cells in these cultures, but NTS, CCK, GLP-1, PYY, and INSL5 secretion could be monitored reliably and simultaneously, through fragments derived from the respective pro-hormones.

As expected from the expression analysis, we could not detect secretion of NTS from distal cultures and INSL5 was only detected in a few samples derived from proximal colon. ProCCK levels were also lower, and PYY3-36 levels higher, in supernatants from distal vs proximal cultures. In proximal colonic cultures, AM1638 stimulated secretion of NTS, proCCK, GRPP (a peptide from *Gcg*) and PYY, whereas AVP and AngII were largely without effect. By contrast, all three GPCR ligands stimulated secretion of proCCK, GRPP, PYY, and INSL5 from the distal cultures, consistent with the enrichment of *Avpr1b* and *Agtr1a* in L^Insl5^, but not L^Nts^ cell clusters. Results from the middle section fell in between the results from the proximal and distal large intestine, both with respect to the levels of individual peptides detected and the responsiveness to the different stimuli.

We next examined whether large intestinal L-cells could be stimulated specifically *in vivo* using agonists of AVPR1B and AGTR1A. Expression of these receptors in bulk-purified L-cells from duodenum, ileum, and colon/rectum was assessed from our published RNA-sequencing data [3], revealing that whereas *Agtr1a* expression was restricted to the distal gut, *Avpr1b* was also expressed in more proximal L-cells in the small intestine (Figure 4B). Mice were injected ip with AVP, AngII or vehicle control, and plasma GLP-1 and PYY levels measured by immuno-assay after 15 min. GLP-1 levels increased ∼2-fold following stimulation with either AVP or AngII (Figure 4I and J). PYY levels were elevated significantly by AVP but not AngII (Figure 4K and L).

## 4 Discussion

In this study, we used scRNA-seq to characterize the EEC landscape of the mouse large intestine. Consistent with previous literature [11] colonic EECs fall into three major groups based on their expression of *Gcg* and *Pyy* (L-cells), *Tph1* (EC-cells) or *Sst* (D-cells). Ecm-cells were the most abundant cells constituting ∼50% of all Ecm-cells, followed by L-cells (∼40%) and the remaining ∼10% being D-cells, matching previous reports.

L-cells and Ecm cells formed several distinct sub-clusters, exhibiting transcriptional profiles consistent with the idea that they differ along the crypt-surface and proximal-distal gut axes. Consistent with previous data from the small intestine [7], [28], *Sct*-positive Ecm-cells expressed lower levels of *Tac1* (encoding the precursor of neurokinin-A and substance-P), compared with other Ecm-cells, whereas *Sct*-positive L-cells expressed lower levels of *Gcg* compared with other L-cells. It is thus likely that the *Sct/Tac* and the *Sct/Gcg* subgroupings in Ecm and L-cells, respectively, distinguish surface epithelial cells from deep crypt cells in the colon and rectum. Consistent with this hypothesis, a previous report showed enrichment of GCG in a deep crypt preparation of the human colon compared with surface epithelium [37], whereas we observed higher expression of cell cycle inhibitors such as *Cdkn1a* in the *Sct*-enriched cells. A physiological reason for this apparent hormonal switch as enteroendocrine cells mature is still unclear.

ScRNA-seq has recently been combined with temporally restricted fluorescent protein expression to map transcriptional changes occurring during EEC differentiation in the mouse small intestine [28]. Mirroring their data, we identified similar transcription factors specifically enriched in L-cells (e.g. *Arx*), Ecm-cells (e.g. *Atf6*) and D-cells (e.g. *Hhex*) from the large intestine, also consistent with our previous reports that *Hhex* is enriched in *Sst*-positive cells in the stomach and pancreas [40], [41]. Our correlation analysis of *Sct*-positive vs negative EEC clusters from the large intestine also revealed higher expression in *Sct*-positive cells of transcription factors that were found by temporal fluorescence mapping to be expressed only late in EEC development in the small intestine [28], e.g. *Id1-3* and *Gtf2f2*. Our results therefore suggest that EEC development and differentiation in the colon and rectum follow similar pathways to those previously described in the small intestine.

In addition to the transcriptomic signatures characteristic of EEC development along the crypt-surface axis, we found that both Ecm cells and L-cells displayed a proximo-distal expression gradient. Distally located L-cells characteristically expressed *Insl5* rather than *Nts* and processed PYY more efficiently to the shorter 3-36 form; the physiological relevance of this hormonal signature change in the distal large intestine remains obscure. It could be speculated that intracellular processing to PYY3-36 enables more selective stimulation of Y2R in the vicinity of L-cells before PYY1-36, which has a broader Y-receptor repertoire [42], is processed by DPP4 expressed in endothelial cells [43]. Ecm-cells by contrast, exhibited a strong longitudinal gradient of *Piezo2*, a mechanosensitive channel previously implicated in small intestinal Ecm-cell stretch sensitivity [44]. It is currently unclear why the most distally located Ecm-cells would need a higher expression of these channels, especially as Ecm-cells seem to be redundant for the initiation of colonic peristalsis and pellet propulsion [45], [46], [47].

EC-cell and L-cell clusters exhibited differential expression of a number of GPCRs. *Olfr78, Olfr558*, and *Ffar2* were more highly expressed in *Tac1*-positive than *Sct*-positive Ecm cells, suggesting these receptors are predominantly located deeper in the crypts rather than the surface epithelium. As these receptors are believed to sense microbially generated short chain fatty acids [48], [49], [50], this raises an interesting question of whether microbiota residing within colonic crypts are physiologically more important for providing signals to EECs than those resident in the lumen. Confirming a previous report examining the expression profile of small intestinal and colonic Ecm cells [50], we also found that GPCRs classically involved in detecting nutrient ingestion in small intestinal L-cells, including *Ffar1*, *Gpr119*, and *Gpbar1*, were expressed in large intestinal L-cell clusters but absent from corresponding Ecm clusters.

L-cells also exhibited differential GPCR expression along the proximo-distal axis, most notably *Agtr1a* and *Avpr1b*, which were more highly expressed in clusters localized to the distal large intestine. These receptor expression profiles were utilized to examine whether targeted activation of distal L-cells would be sufficient to elevate plasma GLP-1 and PYY levels *in vivo*. Further analysis of receptor expression in the small intestine, however, revealed that whereas *Agtr1a* was indeed restricted to large intestinal L-cells, *Avpr1b* was also found in small intestinal L-cells. *In vitro*, we showed that AVP and AngII triggered hormone release from rectal but not proximal colonic primary cultures. *In vivo*, we were restricted to available immunoassays, as we are currently unable to detect gut hormones at endogenous levels in mouse plasma by LC-MS. Both AVP and AngII elevated plasma GLP-1 levels approximately 2-fold, whereas plasma PYY was elevated by AVP but not AngII. We noted, however, that total PYY levels were relatively high in these plasma samples, perhaps because the immuno-assay employed polyclonal antibodies that would also detect common PYY degradation products [51], [52], making it more difficult to pick up small increments in PYY secretion from plasma measurements. We speculate that AVP was the stronger PYY stimulant because it targeted some small intestinal L-cells in addition to those in the rectum.

The response to AngII injection in mice indicates that targeted stimulation of L-cells in the distal colon and rectum is sufficient to elevate plasma GLP-1 levels *in vivo*. Interestingly, these distal L-cells characteristically expressed *Insl5* alongside *Gcg* and *Pyy*. We previously described INSL5 to have orexigenic properties [24], contrasting with the anorexigenic actions of co-released PYY and GLP-1 (Figure 4) [25]. Whilst the elevated GLP-1 levels following distal L-cell stimulation are likely sufficient to exert a stimulatory effect on insulin secretion, further studies will be required to establish the net effect on food intake of targeting this distal L-cell population.

## Contribution statement

LJB, PL, JL, DAG, and RGK performed experiments, collected and analyzed data. BL and GSHY were involved in initial scRNAseq data analysis, subsequently refined by LJB and PL. AL and JL provided NeuroD1-Cre mice. PL, FMG, and FR wrote the manuscript and all authors contributed to the final version. FMG and FR guarantee the work.

## Appendix A Supplementary data

Fig. S1 **Differently expressed GPCRs and transcription factors**: (A) Heat-map of log_2_ normalized UMIs for each cell for the top differentially expressed genes of the Nts and Insl5 L-cell sub-clusters, D-cells, Secretin, Tac1 and Piezo2 Ecm cell sub-clusters. The top 5 differentially expressed genes between each group and the rest of the cells were identified by a Wilcoxon rank test. (B,C) Heat-map of log_2_ normalized UMI of each cell for the differentially expressed GPCRs (B) and transcription factors (C) for each colonic EEC sub-cluster. (D) Heat-map of log_2_ normalized UMIs for each cell for the top differentially expressed genes of the four L-cell sub-clusters. The top 5 differentially expressed genes between each group and the rest of the cells were identified by a Wilcoxon rank test. (E.F) Heat-map of log_2_ normalized UMI of each cell for the differentially expressed GPCRs (E) and transcription factors (F) for each colonic L-cell sub-cluster. Fig. S1
