# Supplementary figures and images for: Single cell transcriptomic profiling of large intestinal enteroendocrine cells in mice – Identification of selective stimuli for insulin-like peptide-5 and glucagon-like peptide-1 co-expressing cells
Source: Mol Metab. 2019 Sep 7;29:158–69. doi: 10.1016/j.molmet.2019.09.001 (PMC6812004; doi:10.1016/j.molmet.2019.09.001)

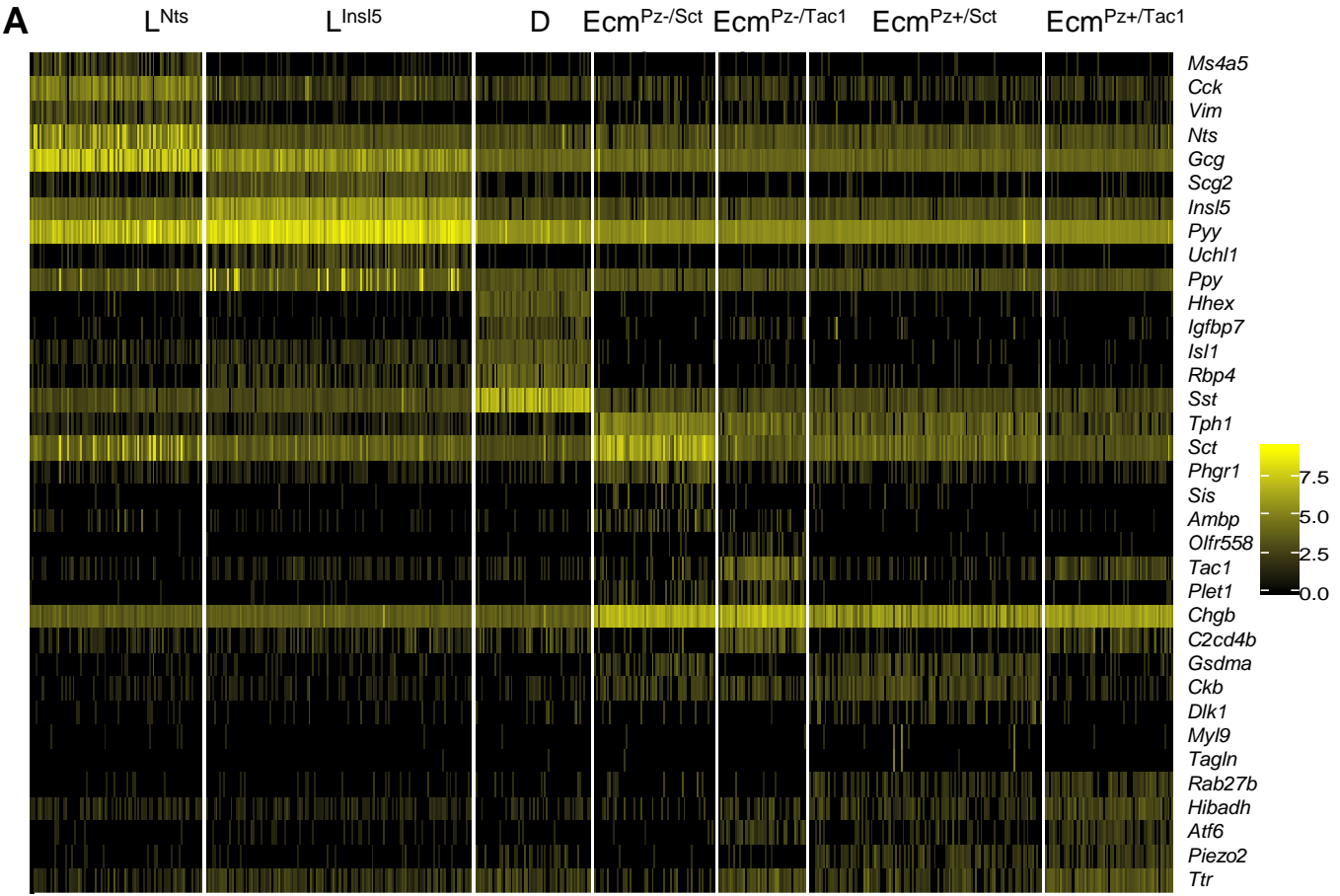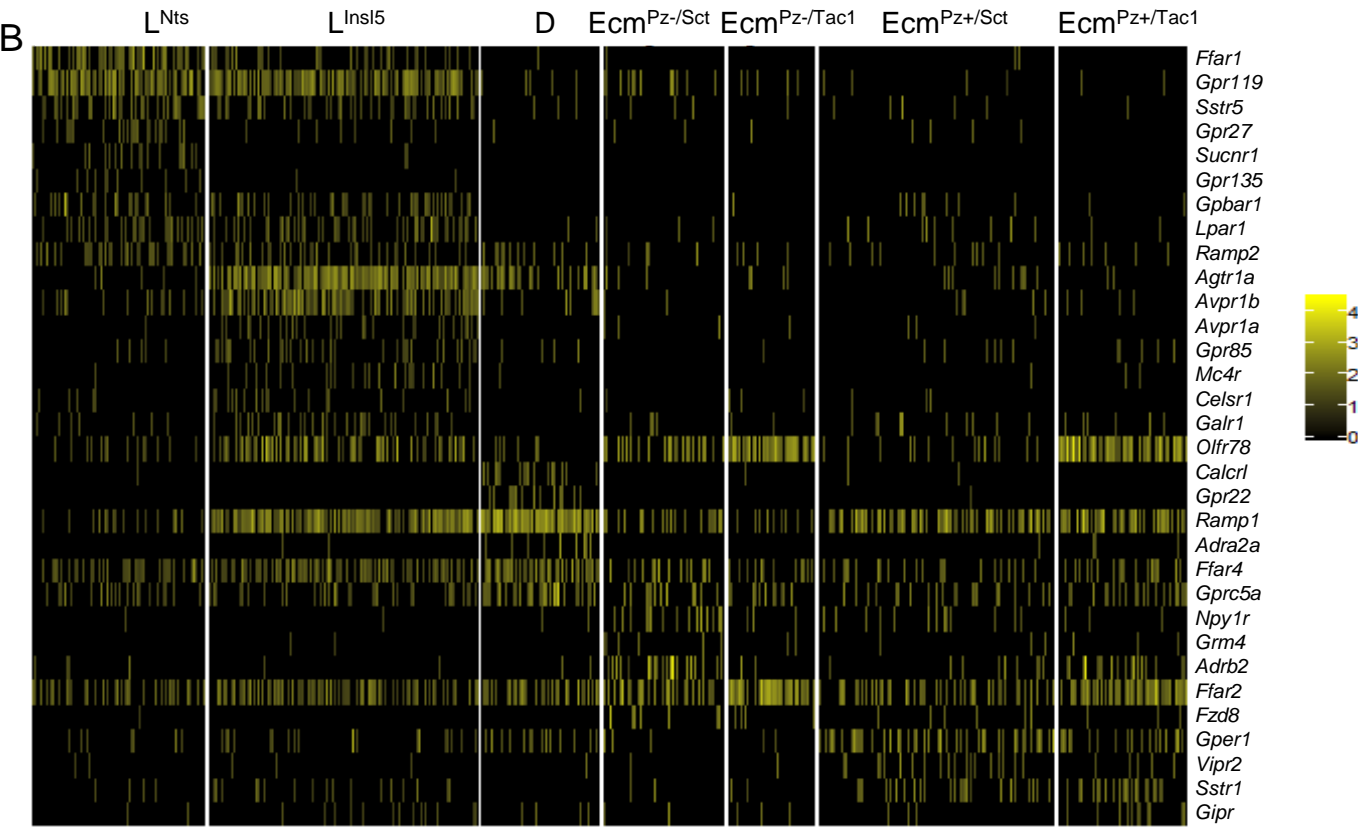

C

L<sup>Nts</sup>

L<sup>InsI5</sup>

D

Ecm<sup>Pz-/Sct</sup>

Ecm<sup>Pz-/Tac1</sup>

Ecm<sup>Pz+/Sct</sup>

Ecm<sup>Pz+/Tac1</sup>

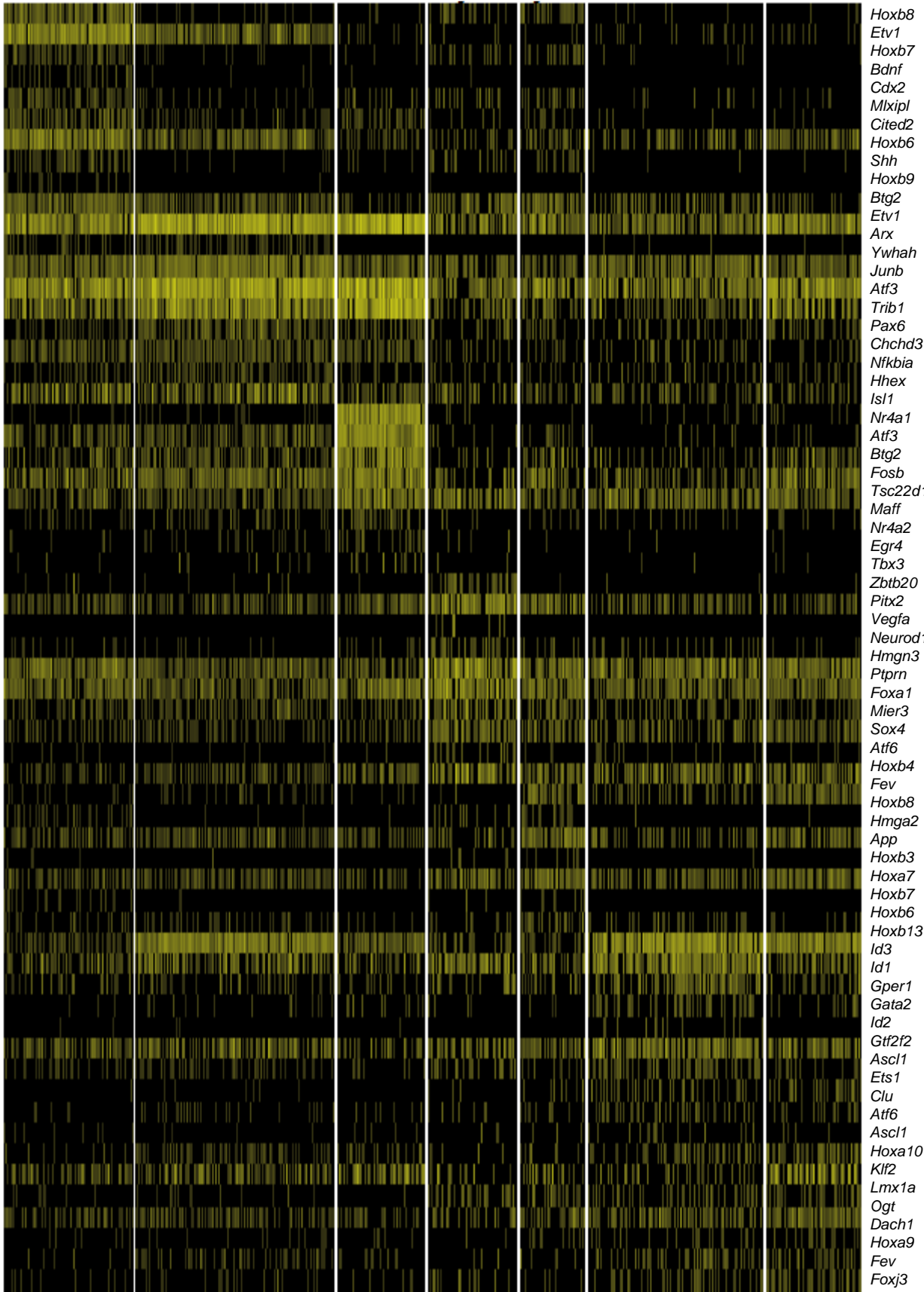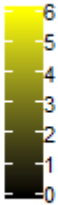

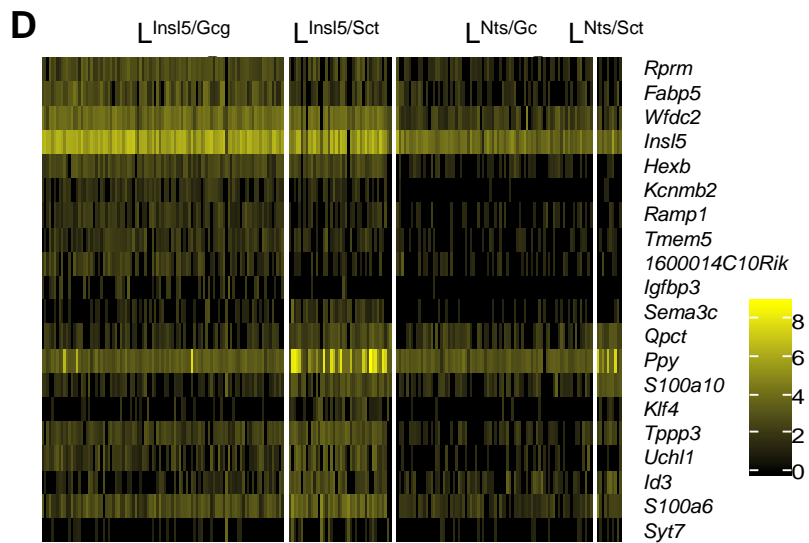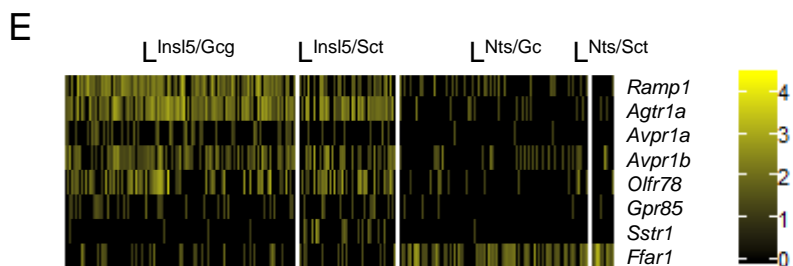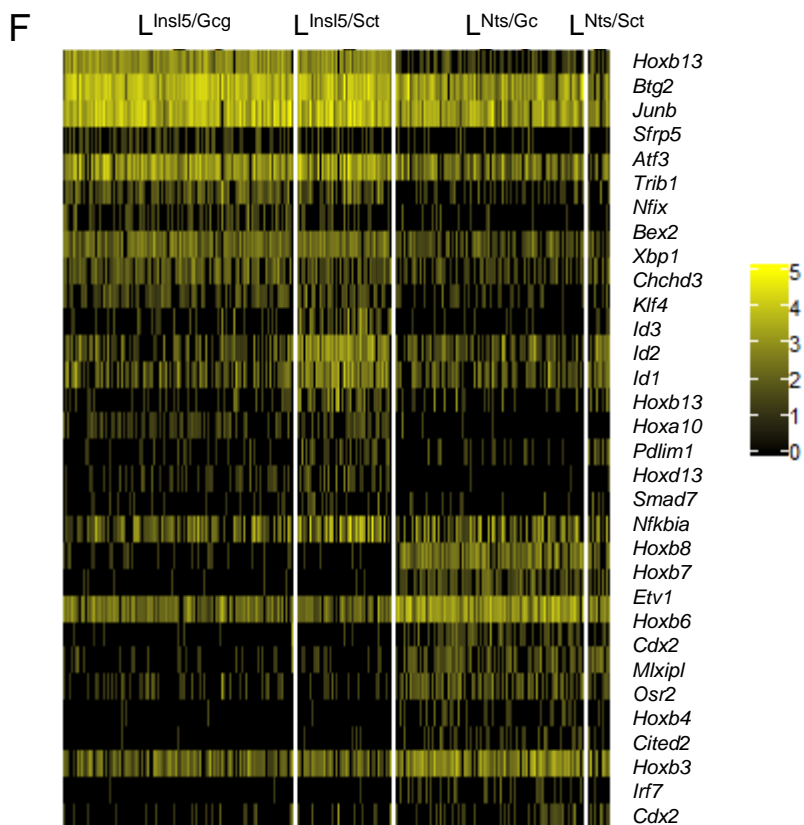

Supplement: Fig. S1 — Differently expressed GPCRs and transcription factors: (A) Heat-map of log2 normalized UMIs for each cell for the top differentially expressed genes of the Nts and Insl5 L-cell sub-clusters, D-cells, Secretin, Tac1 and Piezo2 Ecm cell sub-clusters. The top 5 differentially expressed genes between each group and the rest of the cells were identified by a Wilcoxon rank test. (B,C) Heat-map of log2 normalized UMI of each cell for the differentially expressed GPCRs (B) and transcription factors (C) for each colonic EEC sub-cluster. (D) Heat-map of log2 normalized UMIs for each cell for the top differentially expressed genes of the four L-cell sub-clusters. The top 5 differentially expressed genes between each group and the rest of the cells were identified by a Wilcoxon rank test. (E.F) Heat-map of log2 normalized UMI of each cell for the differentially expressed GPCRs (E) and transcription factors (F) for each colonic L-cell sub-cluster. [file mmc1.pdf]
